# Increased Facial Attractiveness Following Moderate, but not High, Alcohol Consumption

**DOI:** 10.1093/alcalc/agv010

**Published:** 2015-02-25

**Authors:** Jana Van Den Abbeele, Ian S. Penton-Voak, Angela S. Attwood, Ian D. Stephen, Marcus R. Munafò

**Affiliations:** 1 School of ExperimentalPsychology, University of Bristol, Bristol, UK; 2 MRC Integrative Epidemiology Unit (IEU) at the University of Bristol, Bristol, UK; 3 UK Centre for Tobacco and Alcohol Studies, University of Bristol, Bristol, UK; 4 Department of Psychology, Macquarie University, Sydney, Australia

## Abstract

**Aims:** Alcohol consumption is known to be associated with risky sexual behaviours, but this relationship may be complex and bidirectional. We explored whether alcohol consumption leads to the consumer being rated as more attractive than sober individuals.

**Methods:** Heterosexual social alcohol consumers completed an attractiveness-rating task, in which they were presented with pairs of photographs depicting the same individual, photographed while sober and after having consumed alcohol (either 0.4 or 0.8 g/kg), and required to decide which image was more attractive.

**Results:** Photographs of individuals who had consumed a low dose of alcohol (equivalent to 250 ml of wine at 14% alcohol by volume for a 70 kg individual) were rated as more attractive than photographs of sober individuals. This was not observed for photographs of individuals who had consumed a high dose of alcohol.

**Conclusion:** In addition to perceiving others as more attractive, a mildly intoxicated alcohol consumer may also be perceived as more attractive by others. This in turn may play a role in the relationship between alcohol consumption and risky sexual behaviour.

## INTRODUCTION

Evolutionary psychologists have suggested that physical attractiveness serves as a signal that facilitates the identification of healthy, high-quality mates (Fink and Penton-Voak, 2002; Rhodes *et al.*, 2007). Observable traits such as facial attractiveness can act as a cues to mate quality (genotypic and/or phenotypic); for example, facial symmetry, averageness and sexual dimorphism have all been suggested to reflect physical health and viability, which may be heritable (Penton-Voak and Perrett, 2000). Bilateral symmetry is hypothesized to reflect developmental stability, signalling an ability to cope with environmental stressors and withstand parasitic and pathogenic insults (Fink and Penton-Voak, 2002; Weeden and Sabini, 2005; Rhodes *et al.*, 2007), while both symmetry and averageness have been proposed to positively correlate with heterozygosity and hence may signal one's genetic diversity in defence against parasites (Fink and Penton-Voak, 2002). Feminine traits in women, and, in particular, masculine physical traits in men may also advertise effective immune systems, as sex-specific hormones are thought to be immune-suppressive (Jones *et al.*, 2005; Weeden and Sabini, 2005) (although see (Scott *et al.*, 2013) for an alternative viewpoint). These three traits have all been associated with attractiveness judgements in faces (Weeden and Sabini, 2005; Rhodes, 2006).

Facial attractiveness is associated with various measures of health, supporting the view that certain elements of facial attractiveness have been sexually selected due to their signalling value (Rhodes, 2006). However, while facial shape—symmetry, averageness, sexual dimorphic traits—has been extensively studied, only relatively recently have studies investigated the potential role of skin texture and colouration (Penton-Voak *et al.*, 2001; Jones *et al.*, 2004; Stephen *et al.*, 2009a). Jones and colleagues (Jones *et al.*, 2004) reported that health ratings of skin patches were correlated with ratings of attractiveness for the male faces from which the patches were extracted. In addition, composite images with ‘healthy-looking skin’ were also found to be more attractive than ‘unhealthy skin’ composites, independent of shape (Jones *et al.*, 2004). Skin colour distribution, topography and homogeneity have since all been related to both health and attractiveness (Fink *et al.*, 2006; Matts *et al.*, 2007; Fink and Matts, 2008).

Two recent studies (Stephen *et al.*, 2009a,b) investigated the relationship between skin colour and health perceptions. Testosterone for example, has been shown to promote peripheral vasodilation in men, increasing blood flow to visible areas of skin. Red colouration could thus signal physiological strength, since it incurs energetic and immunosuppressive costs (Elliot *et al.*, 2010). In women, increased sex hormone levels are associated with increased skin vascularization and vasodilatory responses (Thornton, 2002). Greater redness of the skin can also be produced by higher levels of oxygenated haemoglobin in the blood, a trait associated with increased aerobic fitness. Increased blood deoxygenation, on the other hand, is often indicative of respiratory or cardiac illnesses (Stephen *et al.*, 2009a,b; Elliot *et al.*, 2010). Skin blood perfusion and oxygenation thus relate to hormonal and cardiovascular health, and have been shown to act as socio-sexual signals of dominance, physiological health and reproductive status in non-human primates, making them strong potential cues for mate choice (Stephen *et al.*, 2009a).

When participants were asked by Stephen and colleagues (Stephen *et al.*, 2009a) to optimize the healthy appearance of facial images by manipulating them simultaneously along oxygenated and deoxygenated blood colour axes, deoxygenated blood colour was decreased and oxygenated blood was increased. The combined colour change led to an amplification of overall redness and a small reduction in overall blueness, which indicated that the participants preferred a skin colour consistent with elevated blood perfusion and oxygenation. In another study, participants were asked to manipulate skin colour along colour axes to enhance healthy appearance. In addition to increasing skin yellowness and lightness, participants also increased skin redness, again supporting the idea that skin colour may be an important fitness indicator (Stephen *et al.*, 2009b).

Alcohol consumption has a vasodilatory effect, causing mild flushing following consumption (Kupari *et al.*, 1983; Paton, 2005). It may also causes changes in the appearance of the face in other ways, such as changes in facial expression due to changes in mood, sexual arousal and sex expectancy (George and Stoner, 2000). For example, facial expressions of happiness (i.e. smiling), have been shown to enhance ratings of attractiveness (Mueser *et al.*, 1984; O'Doherty *et al.*, 2003), particularly for women's faces (Penton-Voak and Changj, 2008). Alcohol consumption is known to be associated with sexual behaviour in social settings (and in particular risky sexual behaviour) (Cooper, 2002), and it is therefore important to understand the mechanisms through which alcohol might influence these behaviours. Previous studies have shown that alcohol consumption leads to an increase in the rating of attractiveness of others (Jones *et al.*, 2003; Parker *et al.*, 2008; Attwood *et al.*, 2012; Chen *et al.*, 2014). However, the direct effects of alcohol on the perceived attractiveness of the *consumer* have not, to our knowledge, been systematically investigated. Given the relationship between facial characteristics such as colouration and perceived attractiveness, and the potential impact of alcohol consumption on these characteristics, it is logical to explore whether alcohol consumption alters the perceived attractiveness of the consumer.

In this study, we sought to explore the possibility that alcohol consumption leads to the consumer being rated as more attractive than sober individuals. In other words, we asked whether consumption of moderate amounts of alcohol enhances the attractiveness to the opposite sex *of the consumer*.

## METHODS

### Participants

Heterosexual social alcohol consumers were recruited from students at the University of Bristol. Participants were required to typically consume between 10 and 50 units of alcohol per week for males and 5–35 units per week for females (based on self-report). Alcohol consumption limits were based on United Kingdom government guidelines, and intended to ensure that we only administered alcohol to those who regularly consume it, and not to individuals who may be at risk of problem drinking. All participants were also required to be in good physical and psychiatric health, to not be using illicit drugs (except cannabis), and not to have volunteered for the stimulus collection phase (described below). The study was approved by the Faculty of Science Research Ethics Committee.

### Materials

A total of 104 photographic facial images, 50 of them depicting females, were employed as stimuli in the attractiveness-rating task. These were selected from photographs that had previously been collected using a Canon EOS300D at a focal length of 50 mm under standardized lighting conditions. The images were captured at a resolution of 2048 by 3072 pixels, in full colour. For each photograph, the camera lens was positioned at the same level as the eyes, thus providing replicable images for each individual (Parker *et al.*, 2008), and for each individual the camera settings were held constant to ensure consistent colour between the before and after images. The images were of volunteers who were required to typically consume between 10 and 50 units of alcohol per week for males and 5–35 units per week for females (based on self-report), and were recruited from students at the University of Bristol. They were photographed frontally, with a neutral expression, standing at a distance of ∼1.50 m. Every individual was the heterosexual partner of an individual in the opposite-sex face set, in order to approximately match the attractiveness of the male and female stimuli given the strong correlations observed between the attractiveness of romantic partners (Feingold, 1988). Each volunteer was photographed three times: (a) when sober, (b) after the consumption of 0.4 g/kg of alcohol and (c) after the consumption of a further 0.4 g/kg of alcohol (i.e. a total dose of 0.8 g/kg of alcohol), while the room temperature was held constant.

The questionnaire measures used included the Positive Affect and Negative Affect Scale (PANAS) (Watson *et al.*, 1988), the Sociosexual Orientation Index—Revised (SOI-R) (Penke and Asendorpf, 2008) and visual analogue scales (VAS) of mood, comprising the terms ‘happy’, ‘drowsy’, ‘depressed’, ‘nervous’, ‘energetic’, ‘irritable’ and ‘sexually aroused’ rated on a 100 mm scale from ‘Not at all’ to ‘Extremely’.

### Procedure

Following screening for eligibility and informed consent, recent alcohol consumption was tested using a breath alcohol monitor. Participants were then asked to complete baseline rating of mood (PANAS, VAS), after which they completed the attractiveness-rating task, which was self-paced. They were sequentially presented with pairs of photographs (400 × 504 pixels) depicting the same individual, comprising either the sober and low alcohol images, or sober and high alcohol images. Participants were required to decide which image was more attractive, and to what extent, using the number keys 1–8 on the testing computer. Values 1–4 indicated that the left presented face was preferred (1 = *strongly prefer*, 2 = *prefer*, 3 = *slightly prefer*, 4 = *guess*), while values 5–8 indicated that the right presented face was preferred (5 = *guess*, 6 = *slightly prefer*, 7 = *prefer*, 8 = *strongly prefer*). The images were displayed on a monitor, which was colour-calibrated using ColorVision Spyder 2Pro. The presentation of the stimuli was controlled by E-Prime v.1.2 software (Psychology Software Tools, Inc., Pittsburgh, PA, USA). The participants then completed further self-reported measures of mood (PANAS, VAS) and measures of personality (SOI-R), and debriefed as to the purpose and hypotheses of the study.

### Statistical analyses

Responses were re-coded to create a binary variable reflecting preference, so that scores from 1 to 4 indicated a preference for the left presented face, and scores from 5 to 8 reflected a preference for the right presented face. Data were analysed using mixed-model ANOVA of preference and rating data, with condition (low dose, high dose) and participant sex (male, female) as between-subjects factors, and image sex (male, female) as within-subjects factors. These analyses were followed up with one-sample *t*-tests where appropriate, against a value of 50% for the preference data and 4.5 for the rating data (each value reflecting equal preference or rating for the intoxicated and sober faces).

In order to investigate the impact of alcohol on facial skin colour, MATLAB (MathWorks, Natick, MA, USA) was used to determine the average colour of the skin portions of the photographic facial images used in the attractiveness-rating task. Colour was measured in the CIELab colour space, which is based on the human colour visual system and designed to be perceptually uniform (such that a change of 1 unit along one colour axis is of the same perceptual magnitude as a change of 1 unit along any other colour axis). It is defined by L* (luminance), a* (red-green) and b* (blue-yellow) colour axes. Repeated-measures ANOVA of colour data (L*, a* or b*) was used to test for differences in skin colour between sober, low alcohol and high alcohol conditions.

We calculated that a sample size of 20 participants in the low dose condition and 20 in the high dose condition would provide 80% power at an alpha level of 5% to detect an effect size of *d* = 0.91 for the main effect of condition, and an effect size of *d* = 0.66 for the corresponding one-sample *t*-test within each dose condition. All analyses were conducted using SPSS Statistics version 21.

## RESULTS

### Characteristics of participants

Participants (*n* = 40, 50% female) were aged between 18 and 30 years (mean 20 years, SD 2 years). There were no substantial differences in mean age or SOI-R score between males and females, or between participants randomized to the low-dose or high-dose conditions, no substantial differences in PANAS positive or negative affect, and no evidence for a change in positive or negative affect from baseline to end of testing. These data are presented in Table 1. Similar results were obtained for VAS mood ratings (data not shown).

**Table 1.** Characteristics of participants

	Low dose (*n* = 20)			High dose (*n* = 20)		
	Males (*n* = 10)	Females (*n* = 10)	Combined	Males (*n* = 10)	Females (*n* = 10)	Combined
Age	21.4 (3.3)	20.2 (1.7)	20.8 (2.6)	19.8 (1.6)	19.8 (1.1)	19.8 (1.4)
SOI-R	36.30 (7.23)	34.30 (9.70)	35.30 (8.41)	32.80 (10.01)	34.50 (15.78)	33.65 (12.89)
	Baseline					
Positive affect	31.2 (7.8)	23.8 (5.7)	27.5 (7.7)	27.8 (7.4)	25.5 (6.4)	26.7 (6.9)
Negative affect	11.9 (1.7)	14.0 (7.7)	13.0 (5.5)	13.1 (7.9)	13.1 (2.7)	13.1 (3.3)
	End of testing					
Positive affect	30.5 (9.8)	21.9 (6.6)	26.2 (9.3)	27.4 (8.3)	24.5 (6.2)	26.0 (7.3)
Negative affect	11.3 (1.6)	12.7 (6.2)	12.0 (4.5)	12.5 (3.4)	11.6 (2.0)	12.1 (2.7)

Values represent mean. Standard deviations are shown in parentheses. SOI-R: Sociosexual Orientation Index—Revised.

### Preference for intoxicated vs sober faces

There was evidence of a main effect of condition on preference for the intoxicated face over the sober face (*F* [1, 36] = 6.96, *P* = 0.012, $\eta_{p}^{2}=0.16$), with preference for intoxicated faces higher in the low-dose condition than in the high-dose condition. Further analyses indicated that this reflected a slight preference for the intoxicated face over the sober face in the low-dose condition (mean preference 54%, 95% CI 50–59%, *P* = 0.057), but a slight tendency to prefer the sober face over the intoxicated face in the high-dose condition (mean preference 47%, 95% CI 43–51%, *P* = 0.097). There was no evidence for any other main effects or interactions (*P*s > 0.31), suggesting that participant and image sex did not influence these results. These data are presented in Table 2.

**Table 2.** Preference for and ratings of intoxicated vs sober faces

	Low dose (*n* = 20)			High dose (*n* = 20)		
	Male faces	Female faces	Average	Male faces	Female faces	Average
	Preference					
Males (*n* = 20)	0.532 (0.098)	0.520 (0.206)	0.526 (0.116)	0.494 (0.154)	0.463 (0.115)	0.478 (0.098)
Females (*n* = 20)	0.558 (0.106)	0.560 (0.155)	0.559 (0.066)	0.439 (0.130)	0.469 (0.115)	0.454 (0.075)
Combined (*n* = 40)	0.545 (0.100)	0.540 (0.178)	0.542 (0.093)	0.466 (0.141)	0.466 (0.112)	0.466 (0.086)
	Ratings					
Males (*n* = 20)	4.638 (0.354)	4.693 (0.725)	4.667 (0.370)	4.556 (0.690)	4.452 (0.503)	4.506 (0.417)
Females (*n* = 20)	4.763 (0.403)	4.799 (0.441)	4.784 (0.191)	4.211 (0.543)	4.365 (0.503)	4.274 (0.381)
Combined (*n* = 40)	4.701 (0.375)	4.746 (0.587)	4.723 (0.293)	4.384 (0.630)	4.409 (0.491)	4.390 (0.407)

Values represent proportion (preference) and mean (ratings). Standard deviations are shown in parentheses.

### Ratings of intoxicated vs sober faces

A similar pattern was observed for rating data, with evidence of a main effect of condition (*F* [1, 36] = 8.69, *P* = 0.006, $\eta_{p}^{2}=0.20$). This reflected higher ratings for the intoxicated face over the sober face in the low-dose condition (mean difference +0.22, 95% CI +0.09 to +0.36, *P* = 0.003), but no clear difference in ratings for the intoxicated face over the sober face in the high-dose condition (mean difference −0.10, 95% CI −0.29 to +0.09, *P* = 0.264). There was no evidence for any other main effects or interactions (*P*s > 0.14), suggesting that participant and image sex did not influence these results. These data are presented in Table 2.

### Colour changes in intoxicated vs sober faces

For the redness (a*) axis, there was evidence of a main effect of alcohol level (*F* [2, 52] = 4.35 *P* = 0.018, $\eta_{p}^{2}=0.14$). Bonferroni-corrected pairwise comparisons indicated that faces in the low alcohol condition were redder (higher a*) than faces in the sober condition (mean difference −0.51, 95% CI −0.96 to −0.05, *P* = 0.026). However, there was no clear evidence of a difference between sober and high alcohol (mean difference −0.21, 95% CI −0.67 to +0.26, *P* = 0.80) or low alcohol and high alcohol (mean difference +0.30, 95% CI −0.10 to + 0.70, *P* = 0.20) conditions.

For the luminance axis, a main effect of alcohol condition was also observed (*F* [2,52] 4.94, *P* = 0.011, $\eta_{p}^{2}=0.16$). Bonferroni-corrected pairwise comparisons indicated that faces were darker (lower L*) in the low alcohol than in the sober condition (mean difference +1.00, 95% CI +0.15 to +1.86, *P* = 0.018). There was no clear evidence of a difference between the sober and high alcohol (mean difference +0.61, 95% CI −0.20 to +1.41, *P* = 0.19) or low alcohol and high alcohol (mean difference −0.39, 95% CI −1.20 to +0.41*, P* = 0.67) conditions.

For the yellowness (b*) axis, no main effect of alcohol condition was observed (*P* = 0.39).

## CONCLUSIONS

Our results suggest that faces of individuals who have consumed a low dose of alcohol (equivalent to 250 ml of wine at 14% alcohol by volume for a 70 kg individual) are rated as more attractive than faces of sober individuals. This was not observed for faces of individuals who had consumed a high dose of alcohol (equivalent to 500 ml of wine at 14% alcohol by volume in a 70 kg individual). These results suggest that the effects of alcohol consumption on risky sexual behaviours may be complex and bidirectional. Previous studies have shown, both observationally and experimentally, that alcohol consumption increases ratings of attractiveness of other people (Jones *et al.*, 2003; Parker *et al.*, 2008; Attwood *et al.*, 2012; Chen *et al.*, 2014). However, the present study suggests that alcohol consumption also increases ratings of attractiveness of the *consumer* by other people. That is, in addition to perceiving *others* as more attractive, an alcohol consumer may also be *perceived* by others as more attractive, and therefore receive greater sexual interest from potential mates. An increase in such attention from others may also positively reinforce alcohol consumption, particularly in social contexts.

The mechanism that leads to this apparent increase in attractiveness is currently unknown, although some possibilities present themselves. Given the nature of our study (using photographic stimuli), the change in attractiveness is presumably driven by changing appearance following alcohol consumption. One possible mechanism is vasodilation associated with alcohol consumption, which may lead to an increase of skin blood perfusion in the skin and an increase in red colouration, which in turn is known to be perceived as healthy (Stephen *et al.*, 2009a,b) and attractive (Stephen *et al.*, 2012). This explanation is consistent with our finding that in the low alcohol condition the skin tone in our facial images was slightly redder (higher a*) and darker (lower L*) than in the sober condition, a colour change consistent with increased skin blood perfusion (Stephen *et al.*, 2009a). In a sense, the action of alcohol on colouration may ‘hijack’ mechanisms designed to promote attraction to healthy mates. However, this difference was not observed between sober and high alcohol or between low alcohol and high alcohol conditions, suggesting that the flushing effect of alcohol—along with the attractiveness enhancing effect of alcohol—may be most pronounced after moderate alcohol consumption. Another possibility is facial expression; low doses of alcohol may lead to an increase in positive mood that is apparent in subtle smiles and relaxation of tonic muscle tone. However, at this stage these possibilities remain speculative and will require further investigation.

One interesting aspect of our data is the curvilinear pattern observed for ratings of attractiveness of faces of individuals who are sober or have consumed low or high amounts of alcohol. For all of our outcome measures (preference, ratings and colour), we observed an effect for faces in the low-dose condition compared with the sober condition, but not the high-dose condition compared with the sober condition. This suggests that any effects of alcohol consumption on the perceived attractiveness of consumers only occur within a relatively narrow window of consumption. It may be that the flushing effects of alcohol consumption are short-lived, and therefore only observed after the consumption of an initial drink. Our colour analysis would support this interpretation, since we did not observe any difference between the sober and high-dose conditions. Alternatively, it may be that changes in facial expression become excessive (and therefore unattractive) after high levels of alcohol consumption. Without data on whether the effects we observed are due to facial expression, we cannot exclude this possibility. These possibilities will therefore require further investigation.

There are a number of limitations to be considered when interpreting the results of this study. First, we cannot say with certainty whether the effects we observed are due to the effects of alcohol on facial colouration, or operate via some other mechanism (e.g. facial expression). While our analysis of colour change supported this as a potential mechanism, our study was not designed to support a formal mediation analysis. We also did not collect data on mood or intoxication, either as perceived in the facial images by participants, or as reported by the consumers themselves during the collection of the photographic facial images. This means we cannot say whether the perceived attractiveness of the consumers is influenced by these factors. These possibilities should be the subject of further investigation. Second, the effects we observed were only observed following low doses of alcohol consumption. It is therefore unclear how strongly these effects might influence behaviour in naturalistic settings, where ratings of attractiveness and sexual behaviour are multiply influenced, and higher doses of alcohol often consumed. Third, the sample we recruited was drawn from a young, student population. While this was in part intentional, given that the facial stimuli used were drawn from a similar population, it would be informative to investigate these effects in a more representative sample. Fourth, while we made careful efforts to ensure that any changes in our stimuli across levels of alcohol consumption were due to the effects of alcohol (for example, by holding room temperature constant), we cannot exclude the possibility that other non-specific factors influenced the composition of these images. However, our colour analysis supports the possibility that the effects we observed are due to alcohol consumption, since the pattern of results is comparable with that observed for ratings of attractiveness. Fifth, this study was exploratory in nature, and the statistical evidence for the observed effects modest. This is unsurprising given that our sample size was only adequate to detect relatively large effects, and therefore lacked power to detect smaller effects. Given the risk that statistically significant findings obtained in underpowered studies are more likely to represent false positives (Button *et al.*, 2013), the findings we report will need to be replicated in a larger sample before they can be considered robust.

In conclusion, our data indicate that alcohol consumption may lead to consumers being rated as more attractive than sober individuals, but only following low levels of consumption. At higher levels of consumption this effect is not observed, and may even be reversed. The increase in skin redness and decrease in skin lightness corresponded to the increase in facial attractiveness in the low alcohol condition, suggesting that facial flushing may drive the increase in facial attractiveness, although a formal mediation analysis was not possible with the current data. Future studies should seek to replicate this finding, and determine whether the effect of low-dose alcohol consumption operates via the colour or shape of the individual, or via some other mechanism. It would also be valuable to explore whether similar results are obtained when ratings are taken in a naturalistic setting in the presence of other cues to attractiveness and sexual behaviour. Understanding the mechanisms through which alcohol influences social behaviour, including factors that may impact on the likelihood of engaging in risky sexual behaviour, is important if we are to develop evidence-based public health messages.

## FUNDING

This work was supported by a European Research Advisory Board grant (EA 08 20) to M.R.M. Funding to pay the Open Access publication charges for this article was provided by the Medical Research Council.

## CONFLICT OF INTEREST STATEMENT

None declared.
